# Increased Throwing Accuracy Improves Children's Catching Performance in a Ball-Catching Task from the Movement Assessment Battery (MABC-2)

**DOI:** 10.3389/fpsyg.2016.01122

**Published:** 2016-07-28

**Authors:** Tim Dirksen, Marc H. E. De Lussanet, Karen Zentgraf, Lena Slupinski, Heiko Wagner

**Affiliations:** ^1^Department of Motion Science, Institute of Sport and Exercise SciencesMünster, Germany; ^2^Institute of Sport and Exercise ScienceMünster, Germany; ^3^Department of Human Performance and Training in Sports, Institute of Sport and Exercise SciencesMünster, Germany

**Keywords:** throwing accuracy, catching performance, MABC-2, children, functional motor tests, catching strategies

## Abstract

The Movement Assessment Battery for Children (MABC-2) is a functional test for identifying deficits in the motor performance of children. The test contains a ball-catching task that requires the children to catch a self-thrown ball with one hand. As the task can be executed with a variety of different catching strategies, it is assumed that the task success can also vary considerably. Even though it is not clear, whether the performance merely depends on the catching skills or also to some extent on the throwing skills, the MABC-2 takes into account only the movement outcome. Therefore, the purpose of the current study was to examine (1) to what extent the throwing accuracy has an effect on the children's catching performance and (2) to what extent the throwing accuracy influences their choice of catching strategy. In line with the test manual, the children's catching performance was quantified on basis of the number of correctly caught balls. The throwing accuracy and the catching strategy were quantified by applying a kinematic analysis on the ball's trajectory and the hand movements. Based on linear regression analyses, we then investigated the relation between throwing accuracy, catching performance and catching strategy. The results show that an increased throwing accuracy is significantly correlated with an increased catching performance. Moreover, a higher throwing accuracy is significantly correlated with a longer duration of the hand on the ball's parabola, which indicates that throwing the ball more accurately could enable the children to effectively reduce the requirements on temporal precision. As the children's catching performance and their choice of catching strategy in the ball-catching task of the MABC-2 are substantially determined by their throwing accuracy, the test evaluation should not be based on the movement outcome alone, but should also take into account the children's throwing performance. Our findings could be of particular value for the development of simple but informative catching assessments, and may provide additional insights into the causes of performance deficits in ball catching.

## Introduction

Ball catching is a fundamental motor skill that is crucial for executing complex movements in various kinds of sport disciplines, thus affecting the overall sport performance. Catching a ball requires visual and motor systems to interact prior to the catching action itself: visual contact with the object is needed, the arm and hand must to be spatially positioned and the fingers extended suitably to the object at the adequate location. Finally, the object must be grasped and saved in the hand. Therefore, a complex and accurate spatiotemporal coordinated movement is necessary to successfully catch a moving object (Williams and McCririe, 1988; Savelsbergh et al., 1993; Peper et al., 1994; Van Waelvelde et al., 2003; Zago et al., 2009; Tijtgat et al., 2010).

The execution of this movement can vary considerably between subjects. They can intercept the ball over a wide range of its trajectory and task-specific time window (Cesqui et al., 2012). Moreover, the abundant degrees of freedom (DoF) of the human body allow for a variety of different catching strategies to achieve the task goal. For example, the hand can be moved perpendicular to the ball's trajectory and snatching it from the trajectory by predicting the ball's future position (Tresilian and Houseman, 2005). Likewise, the hand can be moved along the ball's trajectory by relying on the continuously changing information of the incoming ball (Dessing et al., 2002). In both cases, the accuracy and precision of the catch depend on how individuals are able to adapt their movement to task-specific spatiotemporal constraints, such as the speed of the object and the object's approach direction toward the interception point (Dessing et al., 2005). In this context, it seems obvious that individuals try to reduce the demands on accuracy and precision to increase their rate of task success. This could be achieved by using a strategy where the moving hand is on the ball's trajectory as long as possible, which is frequently the case when the ball is coming straight toward the catcher (Tresilian and Houseman, 2005).

However, individuals may not be able to adequately control the many DoF with regard to the task goal, so that different movement strategies can also result in highly variable catching success. Based on these potential differences in catching performance, which can also arise from gender and age differences (Junaid and Fellowes, 2006; Olivier et al., 2007; Butterfield et al., 2012), ball catching is used as a key task in functional tests for identifying deficits in the motor performance of children.

Several studies attribute performance deficits in ball catching to an insufficient information processing, such as problems in the visual perception or visual-motor integration (Wright and Sugden, 1996; Sigmundsson et al., 1997; Whyatt and Craig, 2013), impairments in the spatio-temporal anticipation (Estil et al., 2002), or the high amount of time needed to select the most important information (Lefebvre and Reid, 1998). Others report on qualitative differences in the movement execution and have shown that various kinematic catching parameters, such as range of motion across joint rotations (Sekaran et al., 2012), wrist trajectories and spatiotemporal distributions of the impact point (Laurent et al., 1994; Cesqui et al., 2012; Asmussen et al., 2014) can vary greatly between subjects. Moreover, it has been found that children with coordination disorders have difficulties in several components: predicting the ball flight, in controlling posture and in coordinating the involved DoFs (Larkin and Hoare, 1992; Van Waelvelde et al., 2004; Przysucha and Maraj, 2014).

In practical settings, such as school, the tests that are used for identifying deficits in ball catching usually quantify the movement outcome, e.g., by counting the number of balls caught. However, taking into account only the outcome does not provide a valid marker of the deficit's origin, i.e., whether and how the deficit is based on visual or motor impairments (Van Waelvelde et al., 2004).

Moreover, as in case of the one-handed ball-catching task of the Movement Assessment Battery for Children (MABC-2, Henderson and Sugden, 1992), it is questionable to what extent the catching *per se* can even be regarded as the main criterion to determine the children's performance.

As the task requires the person to catch a self-thrown ball, it is difficult to find out whether potential performance deficits arise from the children's throwing or catching performance.

In order to analyze the potential relationship between throwing and catching, we examined the influence of throwing performance on the children's catching performance and their choice of catching strategy when they, as in the above-mentioned ball-catching task, have to throw and catch a ball.

We hypothesize that (H1) the throwing accuracy has an effect on the individual catching performance and that (H2) the throwing accuracy influences the choice of catching strategy.

## Materials and methods

### Participants

Based on a call for participation via e-mail and flyer, 18 healthy children and juveniles (12 males, 6 females) aged 11–15 years (12.61 ± 1.61 years) volunteered in this study. Any motor or cognitive limitations were excluded by the self-disclosure of the children and their parents during a preceding interview. The average body height was 1.65 ± 0.12 m and the average body mass was 49.28 ± 11.02 kg. The children and their parents had been informed several days before the study about the procedure. The study was approved by the local ethics committee (no 2015-13-TD). The parents of all participating children signed a written informed consent. The participants received no payment for their participation.

### Experimental procedure

The task was to throw a tennis ball against a wall and catch it with the throwing hand before the ball touched the ground. During ball release, participants had to stand with both feet behind a marking line on the floor at a distance of 2 m from the wall. For catching the ball, participants were allowed to step over the marking line when needed. Furthermore, they were instructed to catch the ball as cleanly as possible, i.e., without using other parts of their body (see manual of the MABC-2, Age Band 3: 11–16 years).

After a demonstration, a short test phase of 5 throws and catches with each hand was carried out immediately prior to test start. After completing the test phase, 10 throws and catches were performed with each hand. The number of correctly performed catches was recorded. Five reflective markers were attached to each hand (2 markers on the wrist and one marker on thumb, index and pinky) and the ball was marked with reflective foil. The trajectories of the hand markers and the ball were recorded with a motion-capture system composed of 10 infrared high-speed cameras using a sampling rate of 400 Hz (Oqus 500, Qualisys AB, Göteborg, Sweden).

### Data analysis

In line with the test manual of the MABC-2, a trial was classified as successful, when participants (1) stood with both feet behind the marking line on the floor at the time of releasing the ball, (2) caught the ball before it touched the ground, and (3) did not use any other part of the body than the hand. Otherwise the trial was classified as missed.

The MABC-2 test manual also contains age-related standard tables that include standard values based on the number of correctly caught balls with each hand (dominant and non-dominant) and the participant's age. Accordingly, we transformed the raw value of correctly caught balls with each hand into a standard value for each participant (MABC_dom, MABC_ndom) that represents his achieved task performance with the dominant and non-dominant hand, respectively. If the resulting standard value was larger than 10 it was rounded up, if it was smaller than 10 it was rounded down. The standard value is based on a distribution with a mean of 10 and a standard deviation of 3 so that two third of the children have a standard value between 7 and 13.

The 3D-trajectories of the reflective markers and the ball were reconstructed (Qualisys Track Manager, version 2.11). Trials in which one or more markers disappeared for more than 50 ms between release and catch were excluded from analysis (15%). Further processing was done using a Matlab script (Matlab Version R2014a, MathWorks, Natic, MA) as follows: Kinematic data were digitally low-passed filtered using a second-order Savitzky–Golay filter over 21 frames (savitzkyGolayFilt function by R. Losada, Version: 1.11.4.4). The ball's parabola was computed using a linear regression of the vertical component of the ball's velocity between 10 and 110 frames after the ball bounced off the wall. The moment when the ball bounced off the wall was characterized as the maximal distance from the releasing point. The moment of catching the ball was defined as the minimal distance between the center of the hand (i.e., average of the 5 markers attached to the hand) and the fitted ball position.

Initially, we examined the throwing accuracy by measuring the mean distance of the ball bounces from the calculated center of the ball bounces (Bounce_dom, Bounce_ndom): The smaller this mean distance, the greater is the throwing accuracy, and vice versa. In this context, we also calculated the bounce angle (Angle_dom, Angle_ndom) of the ball and the ball's velocity (Velocity_dom, Velocity_ndom) as additional throwing parameters that could have an influence on the catching performance.

Then we examined the participant's catching movement by calculating the duration their hand was on the ball trajectory as a measure to determine differences in the individual catching strategy (HoP_dom, HoP_ndom). For each frame in the period between ball release and catch, it was determined whether the fitted parabola of the ball's trajectory intersected with the plane of the hand. For this, the approximate location of the middle finger was calculated. At first it was determined if two of the lines connecting the hand markers intersected with the plane of the ball. If so, it was determined if the parabola crossed the line through these two points of intersection at a location between these two points. If that was the case, the frame counted as “hand on parabola.” The total number of those frames times 1/400 s (the frame rate) was defined as the duration of hand on the ball trajectory. On basis of all successful trials, we averaged the data per variable and applied bivariate correlations and a regression analysis to examine the relationship between the overall catching performance, throwing accuracy and catching strategy. With the exception of two subjects (4 and 10 successful trials), the number of correct catches ranges between 15 and 20.

### Statistics

All statistical procedures were carried out with SPSS (IBM Corp., Version 22.0, Armonk, New York). Initially, we examined the potential impact of gender and age on the results in order to avoid mistakes in the evaluation and interpretation of the data. We found that age has no significant impact on the test results, whereas gender has. However, we assume that these differences can be neglected, as they arise only due to one outlier in the data and because the data between the male and female participants are not directly comparable given the different sample size.

To provide an overview about the confluence of the parameters examined, we then performed an analysis of bivariate correlations between all parameters of the dominant and non-dominant hand (Table 1). As the data regarding the results of the MABC-2 were not normally distributed, we calculated Spearman's Rho instead of Pearson's product moment correlation coefficient and set the significance level for pairwise comparisons to ∝ = 0.05. For all further steps of statistical analysis, significance is indicated as *p*-value and the significance level has also been set to ∝ = 0.05.

**Table 1:** **Outcome of the bivariate correlation analysis for the dominant and non-dominant hand**.

**Bivariate correlations between the variables regarding the dominant hand**					
	**MABC_dom**	**Bounce_dom**	**Angle_dom**	**Velocity_dom**	**HoP_dom**
MABC_dom	1	−0.468^*^	−0.289	−0.286	0.302
Bounce_dom		1	0.449^*^	0.354	−0.361
Angle_dom			1	0.529^*^	−0.585^*^
Velocity_dom				1	−0.472^*^
HoP_dom					1
**Bivariate correlations between the variables regarding the non-dominant hand**					
	**MABC_ndom**	**Bounce_ndom**	**Angle_ndom**	**Velocity_ndom**	**HoP_ndom**
MABC_ndom	1	−0.577^*^	−0.439	−0.399	0.626^**^
Bounce_ndom		1	0.657^**^	0.296	−0.489
Angle_ndom			1	0.362	−0.554^*^
Velocity_ndom				1	−0.682^**^
HoP_ndom					1

*Significant correlations are printed in bold and marked with asterisk*.

**Figure 1:**
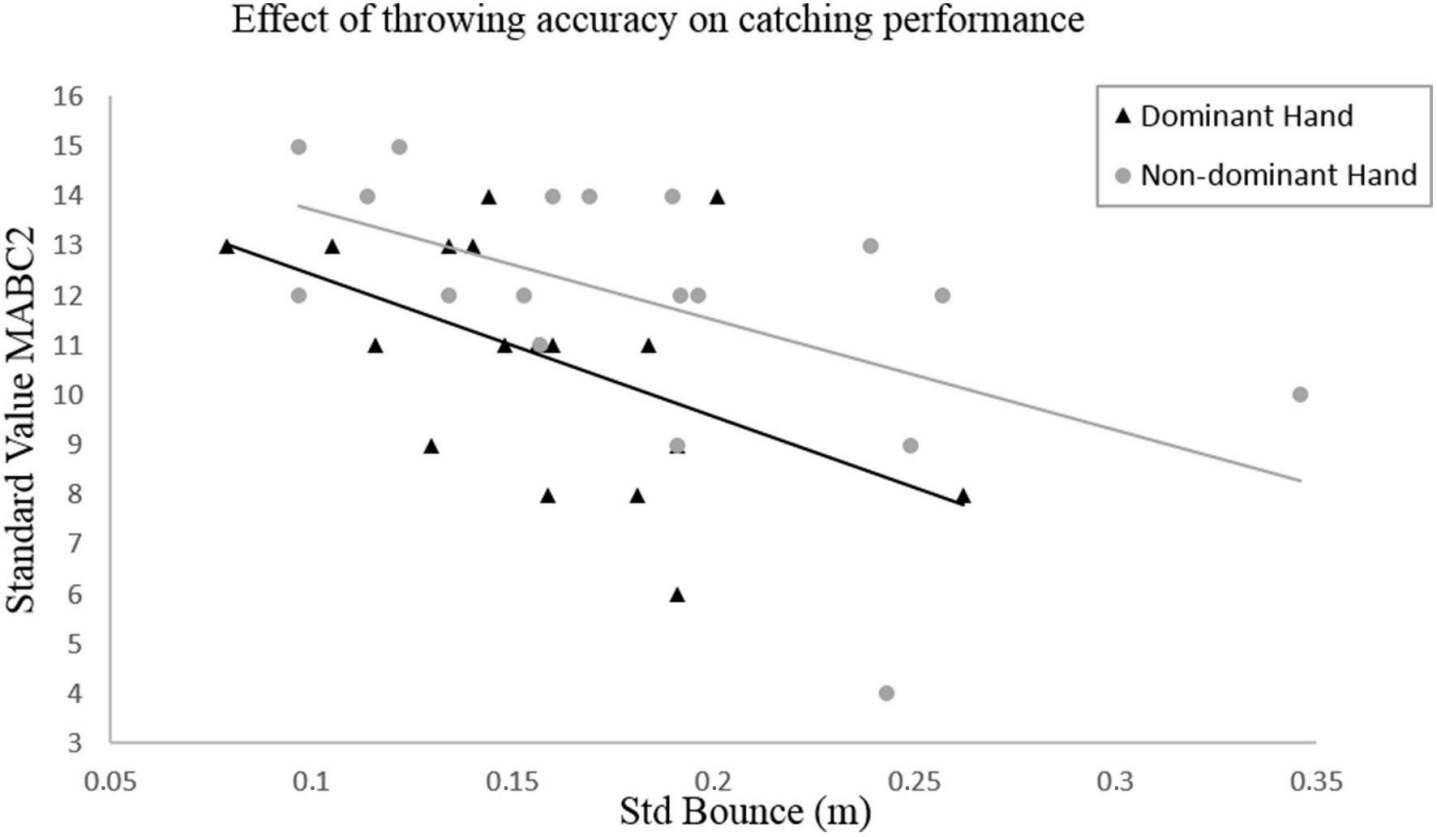
**Bivariate linear regression between the standard deviation of the ball bounce as a measure of throwing accuracy and the standard value of the M-ABC2 as a measure of catching performance**.

**Figure 2:**
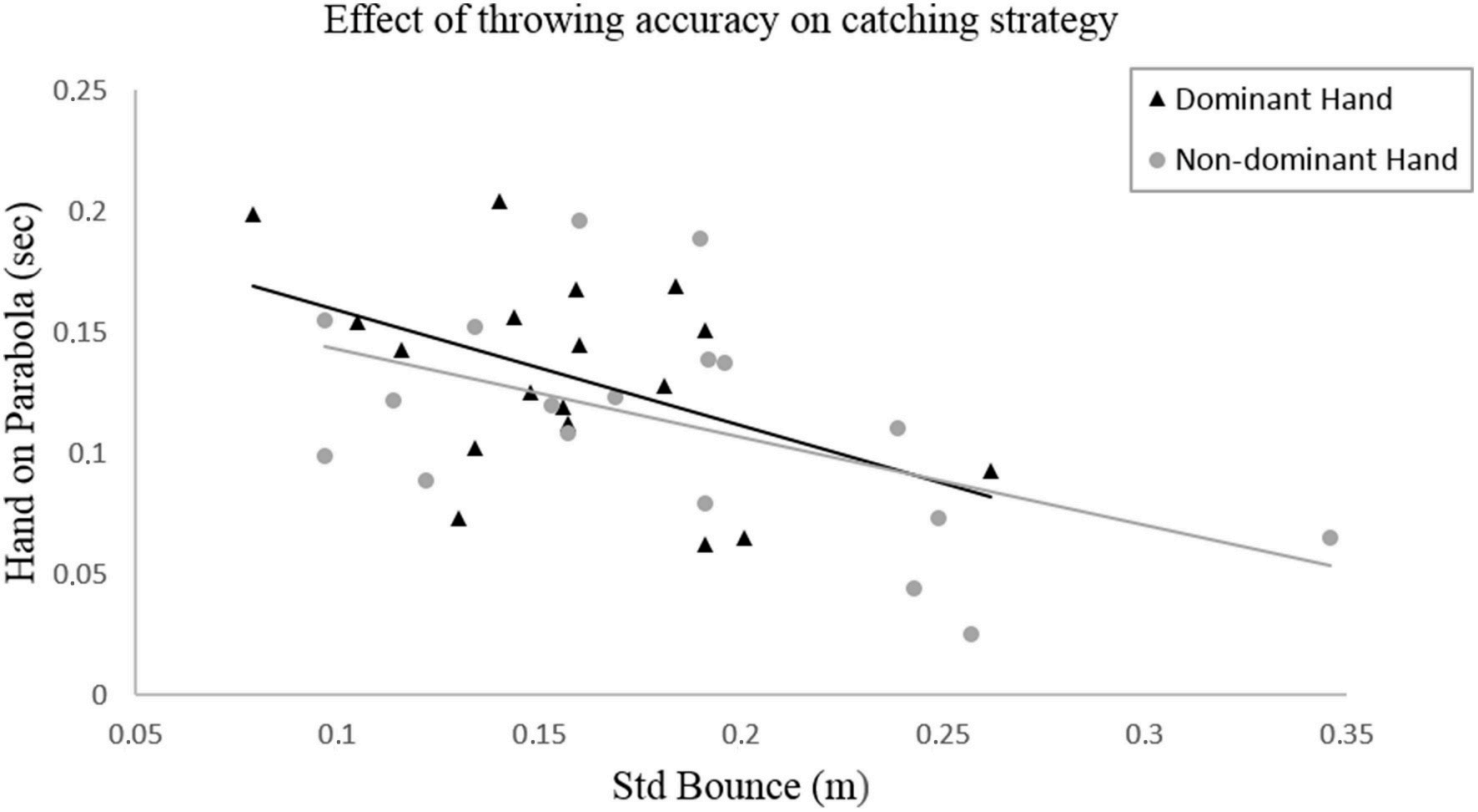
**Bivariate linear regression between the standard deviation of the ball bounce as a measure of throwing accuracy and the duration of the catching hand on the ball's parabola as an indicator of the catching strategy used**.

**Figure 3:**
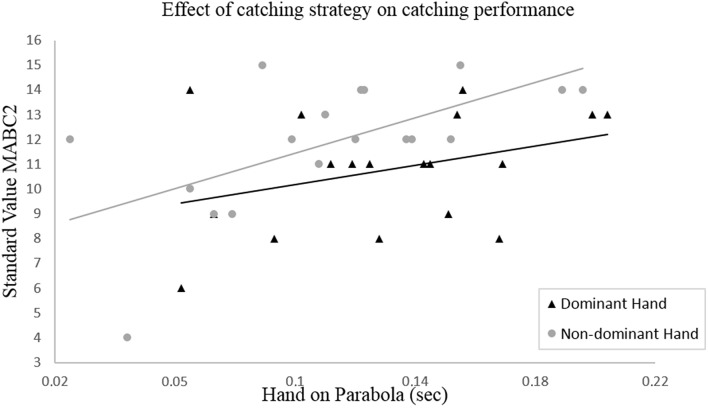
**Bivariate linear regression between the duration of the catching hand on the ball's parabola as an indicator of the catching strategy used, and the standard value of the M-ABC2 as a measure of catching performance**.

Subsequent to the calculation of bivariate correlations, we performed a multiple linear regression analysis by integrating the predictor variables “bounce variation,” “bounce angle,” “ball velocity,” and “hand on parabola” in the model to examine whether the contributions of these variables to the explained variance are independent or not. For those variables that were most suitable for predicting the catching performance, additional bivariate linear regression analyses have been carried out (Figures 1–**3**).

In order to verify the quality of the different linear regression models, we calculated their coefficient of determination (*R*^2^) and examined whether this coefficient was actually based on the relation in the data by looking at the significance level. At the same time, we examined to what extent the requirements for the use of a linear regression analysis have been met. In case of the bivariate regression models, we checked the data for (1) linearity, (2) the same variance of residuals, and (3) the independence of residuals. First, we confirmed visually that linearity in the data exists by plotting the variables of interest in form of simple bivariate regressions. Secondly, it has been shown that the residuals have the same variance, as the bivariate plots of the standardized *y*-values of the regression equation and the standardized residuals contained no recognizable relation. Thirdly, we were able to demonstrate the (relative) independence of the residuals by performing the Durbin-Watson-Test, which yielded values between 1.56 and 2.26.

In case of the multivariate linear regressions, the data were additionally checked for multicollinearity by looking at the tolerance value and the variance inflation factor (VIF). According to Urban and Mayerl (2006), multicollinearity can be excluded when the tolerance value is higher than 0.25 and the VIF is lower than 5. In our study, the tolerance values ranged between 0.36 and 0.72 and the VIF values ranged between 1.39 and 3.06, suggesting that no multicollinearity exists.

## Results

In Table 1 the bivariate correlation matrix for all measured variables is illustrated. For both hands, we found that the bounce variation as a measure of throwing accuracy is significantly correlated with the standard value of the MABC-2 which measures the catching performance. For the non-dominant hand it has also been shown that the variable “hand on parabola” as an indicator for the catching strategy is significantly correlated with the standard value of the MABC-2.

As can be seen from Table 1, there is a relatively high degree of correlation between the predictor variables, which makes it difficult to determine whether their individual variance contributions are independent or redundant. In this context, the multiple linear regression analysis indicates that the predictor variables shared a large proportion of the variance explanation. Particularly in case of the non-dominant hand, the variance explanation is relatively even distributed among all four predictor variables, and the regression model shows a significant relation (*R*^2^ = 0.518, *p* = 0.038).

However, in case of the dominant hand the bounce variation seems to be the best predictor, whereby the regression model with all four predictor variables shows no significant relation (*R*^2^ = 0.288, *p* = 0.315).

Based on the results mentioned above, the aim was to avoid redundancy in the data. Therefore, the following bivariate linear regressions are focused on the variables “bounce variation” and “hand on parabola,” as these are the only factors that have a significant relation with the catching performance. In Figure 1, the relationship between the bounce variation and the catching performance is shown.

Regarding the first hypothesis (H1), the bivariate linear regression analysis reveals a significant relation between the bounce variation and the standard value of the MABC-2 ($R_{dom}^{2}$ = 0.251, *p*_*dom*_ = 0.034; $R_{ndom}^{2}$ = 0.288, *p*_*ndom*_ = 0.021). For both hands, it appears that the catching performance increases when the bounce variation decreases.

In Figure 2 the relationship between the bounce variation and the variable “hand on parabola” is shown.

Regarding the second hypothesis (H2), the bivariate linear regression analysis reveals a significant relation between the bounce variation and the duration the hand is on the ball's parabola ($R_{dom}^{2}$ = 0.218, *p*_*dom*_ = 0.050; $R_{ndom}^{2}$ = 0.265, *p*_*ndom*_ = 0.028). For both hands, it appears that the duration of the hand on the parabola increases when the bounce variation decreases.

This relation is now becoming an important issue because the duration of the hand on the ball's parabola has also an effect on the catching performance. The relationship between the variable “hand on parabola” and the standard value of the MABC-2 is illustrated in Figure 3.

The bivariate linear regression analysis shows that the duration of the non-dominant hand on the parabola is significantly correlated with the standard value of the MABC-2 ($R_{ndom}^{2}$ = 0.376, *p*_*ndom*_ < 0.001). It appears that the catching performance increases when the duration of the hand on the parabola increases as well. In case of the dominant hand, no statistically significant relation was found ($R_{dom}^{2}$ = 0.121, *p*_*dom*_ = 0.156).

## Discussion

The purpose of the current study was to examine whether and to what extent the children's throwing accuracy influences their catching performance and their choice of catching strategy when they have to catch a self-thrown ball.

The results of the bivariate correlations and the linear regression analysis supported the first hypothesis (H1) that the throwing accuracy has an effect on the individual catching performance. For both hands, an increased throwing accuracy in terms of a decreased bounce variation of the ball is significantly correlated with an increased catching performance (Figure 1).

The results also confirmed the second hypothesis (H2) regarding the impact of the throwing accuracy on the choice of catching strategy. For both hands, we found that a higher throwing accuracy is significantly correlated with a longer duration of the hand on the ball's parabola (Figure 2). The main benefit of using this catching strategy can be seen when looking at the children's catching performance achieved with the non-dominant hand, as a longer duration of the hand on the ball's trajectory is reflected in a higher standard value of the MABC-2 (Figure 3). This indicates that throwing the ball more accurately, enables the children to adapt their catching movement to the ball's trajectory more quickly which in turn effectively reduces the requirements on temporal precision (Tresilian and Lonergan, 2002).

For the dominant hand the relation between catching strategy and catching performance does not appear to be significant. The difference between both hands could be due to qualitative differences in the execution of the catching movement that likely arise from varying degrees of experience and skill level. Catching a ball with the non-dominant hand is a relatively unfamiliar movement task so that individuals may not be able to adequately control the involved DoF when they are confronted with different spatiotemporal task constraints, such as varying ball velocities and different approach directions of the ball toward the interception point (Dessing et al., 2005). It is therefore possible that children are required to reduce the temporal constraints of the task by moving their hand on the ball's trajectory as long as possible to compensate potential difficulties with their movement coordination and, at the same time, to increase their rate of task success.

When children catch the ball with the dominant hand, they may not need to reduce the requirements on temporal precision as they are more experienced and higher skilled catchers with this hand, which allows them to intercept the ball's trajectory at many different spatial positions and at varying times without detracting from movement accuracy and catching success.

Another factor that may have influenced the individual catching performance and catching strategy in the present study is the potentially high amount of whole-body movements during the task. Since the children were allowed to move freely after they had released the ball, the task could be performed with an infinite number of individual different movement solutions. This inter-individual variability may have contributed to the fact that some children achieved good catching results even when they threw the ball less accurately or moved their hand along the ball's parabolic trajectory only for a short time. This indicates that the goal of the present ball-caching task can be achieved equally successful with different movement strategies (cf. Cesqui et al., 2012), though we cannot clearly differentiate such movement strategies due to methodological limitations:

Assuming that the children performed each trial of the present task with a certain degree of variability, factors such as the ball's velocity and bounce angle, which may also have an impact on the catching performance, could not be adequately controlled. In addition, the exclusive use of hand kinematics does not allow for drawing valid conclusions on the influence of other involved DoF and the underlying reasons for the different task success.

Another methodological limitation that could have an influence on the data and their interpretation might be that we used a healthy participant population without any motor deficits. As the MABC-2 is designed to detect motor deficits, we can neither exclude a potential ceiling effect in the data, nor can we transpose the results to subjects that actually belong to the target group of the MABC-2.

However, we were able to demonstrate that the children's catching performance and their choice of catching strategy in the ball-catching task of the M-ABC2 are substantially determined by their throwing accuracy. The catching performance in the given task should therefore not be evaluated only by looking at the outcome, but also by considering the throwing accuracy and the entire movement execution. On this basis and in combination with more sophisticated measuring techniques, it might be possible to identify different types of deficits in the interception of moving objects and to derive specific interventions to improve the motor performance in throwing and catching tasks.

## Author contributions

TD, KZ, LS, and HW contributed to the conceptual work; Data acqisition and data analysis were performed by TD, MD; the methods were developed by MD, LS, and HW. TD, KZ, MD, and HW mainly contributed to the writing process of the manuscript. All authors reviewed the final version of the manuscript.

### Conflict of interest statement

The authors declare that the research was conducted in the absence of any commercial or financial relationships that could be construed as a potential conflict of interest.
